# Breeding on a Tropical Island Proves More Difficult Than Expected: The Ongoing Breeding Failure of Laysan Albatross on Clarion Island

**DOI:** 10.1002/ece3.74346

**Published:** 2026-09-30

**Authors:** José Alfredo Castillo‐Guerrero, Sandy Azucena Castañeda‐Castellanos, Daniel Arturo Portillo‐Zavala, Salvador Hernández‐Vázquez, Guillermo Fernández

**Affiliations:** ^1^ Departamento de Estudios Para el Desarrollo Sustentable de la Zona Costera, Centro Universitario de la Costa Sur Universidad de Guadalajara San Patricio‐Melaque Jalisco Mexico; ^2^ Unidad Académica Mazatlán, Instituto de Ciencias del Mar y Limnología Universidad Nacional Autónoma de México Mazatlán Sinaloa Mexico

**Keywords:** breeding success, foraging ecology, nest predation, *Phoebastria immutabilis*, population dynamics

## Abstract

In the 1980s, the Laysan Albatross ( *Phoebastria immutabilis* ) expanded its breeding range into the eastern Pacific and established colonies on several islands off the coast of Mexico. Colony growth has varied widely among these islands, likely reflecting differences in immigration rates and local conditions, such as food availability and predation risk, which can influence breeding success and population dynamics. We assessed the breeding performance of Laysan Albatrosses on Clarion Island (2016–2023) in relation to foraging ecology and local predation. We monitored breeding success through periodic nest checks and quantified predation rates through focal observations. We tracked foraging trips using GPS devices and analyzed blood samples for stable isotopes (*δ* ^15^N, *δ* ^13^C). Over the study period, the colony experienced complete breeding failure. Our results suggest that two factors likely contributed to this outcome: (1) foraging limitations and (2) high predation rates. The remote location of Clarion Island required Laysan Albatrosses to travel farther to feeding grounds, potentially reducing the time available to acquire high‐quality prey. This may have influenced their dietary δ^13^C signatures, which were associated with hatching success in our study. In addition, hatchlings experienced extremely high predation. Together, the apparent foraging constraints and intense local predation hindered successful reproduction and local recruitment. These factors may help explain the limited growth of the Clarion colony over recent decades. This study provides baseline data on the status of the colony and its main threats, which serve as critical input for planning conservation and management strategies.

## Graphical abstract

This study establishes essential baseline data on the current status, colony dynamics, and key threats facing the Laysan Albatrosses on Clarion Island and offers critical insights to guide future conservation and management efforts.

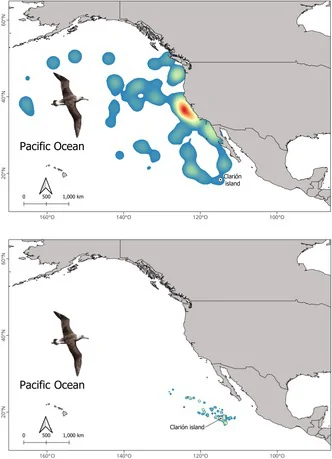

## 1 Introduction

The formation of new seabird colonies is uncommon, especially for philopatric Procellariiformes, and the factors that promote or hinder this process remain poorly understood (Kildaw et al. 2005; Munilla et al. 2016). Initial colonization occurs when immigrants establish a colony, either through natal dispersal by first‐time breeders or breeding dispersal by individuals that have previously bred at another colony. The few studies available on developing colonies indicate that colony establishment can follow contrasting trajectories. Some colonies grow rapidly and become successfully established (Dunlop 2005; Harris and Wanless 1991; Oro and Ruxton 2001), whereas others fail to become established (Storey and Lien 1985) or are eventually abandoned (Brothers 1981). After the initial settlement, new colonies often become highly attractive to potential breeders and typically grow rapidly (Oro and Ruxton 2001; Kildaw et al. 2005; Dunlop 2005; Pyk et al. 2013).

During the 1980s, the Laysan Albatross ( *Phoebastria immutabilis* ) expanded its breeding range from the northeastern Hawaiian Islands to four islands in the eastern Pacific, increasing their reproductive distribution by ~4000 km (Henry III et al. 2021). The first record of Laysan Albatrosses breeding in Mexico occurred on Guadalupe Island in 1983 (Gallo‐Reynoso and Figueroa‐Carranza 1996), followed by records from Clarion Island in 1987 (Howell and Webb 1989), San Benedicto Island in 1990 (Howell and Webb 1992), and Rocas Alijos in 2003 (Pitman et al. 2004). These new colonies may reflect several non‐mutually exclusive factors, including density‐dependent dispersal from established breeding colonies (Pitman 1985; Dunlap 1988) and changes in the eastern Pacific marine ecosystem that may have facilitated range expansion (Henry III et al. 2021). However, these explanations remain speculative, and the mechanisms driving this breeding range expansion remain unclear.

The history of Laysan Albatross colonies in Mexico varies among islands. On Guadalupe Island, colony size has increased rapidly from 1 pair in 1983 (Gallo‐Reynoso and Figueroa‐Carranza 1996) to 1511 pairs in 2020 (Méndez Sánchez et al. 2022), making it the largest colony in the eastern Pacific. The albatrosses breeding on Guadalupe Island forage in the highly productive California Current and expend minimal effort locating food by remaining near foraging sites, taking short trips, and roosting at sea for large portions of their foraging trips (Henry III et al. 2021), which has resulted in high reproductive success and fast colony growth (Henry III et al. 2021; Méndez Sánchez et al. 2022). In addition to the favorable conditions provided by the proximity to foraging areas, the success of this species on Guadalupe Island has also been promoted by targeted management and conservation actions, including feral cat management and the installation of a perimeter fence to protect the breeding colony from terrestrial predators and other disturbances (Hernández‐Montoya et al. 2014; Méndez Sánchez et al. 2022). In contrast to Guadalupe Island, data on other Laysan Albatross colonies in the eastern Pacific are limited. Based on counts of adults or nests, the colonies on other Mexican islands are notably smaller (< 50 nests) and show limited growth compared with the Guadalupe Island colony (Howell and Webb 1989, 1992; Pitman and Ballance 2002; Pitman et al. 2004; Wanless et al. 2009).

Differences in post‐colonization dynamics reflect the attributes and resources available to the Laysan Albatrosses on each island. To understand which factors promote or hinder successful colonization, it is essential to describe these attributes and evaluate how they affect colony growth in different colonization scenarios. In seabirds, food availability and quality, as well as the effort required to obtain it, are crucial factors that influence breeding performance (e.g., Sorensen et al. 2009; Guillemette et al. 2018; González‐Medina et al. 2018). Additionally, nesting site features, such as suitable microclimates for eggs and chicks (Cleeland et al. 2020), parasite control (Espinaze et al. 2020), or protection from predators (Droguett et al. 2023; Degot et al. 2024), can also greatly impact reproductive success.

Clarion Island is part of the Revillagigedo Archipelago, a marine protected area designated by Mexico in the Eastern Tropical Pacific (DOF 2017). It is the most remote island in the archipelago and hosts breeding populations of several bird species, including the Laysan Albatross (Figure 1). The small Clarion Island colony has contained few nests and shown little to no colony growth for decades. To identify the limiting factors affecting this small colony, we assessed the breeding success, foraging ecology, and vulnerability of eggs and hatchlings to predation in Laysan Albatrosses breeding on Clarion Island using GPS tracking, stable isotope analysis, and focal observations. Our findings provide a baseline assessment of the status and threats to Laysan Albatrosses at this site and offer essential data for guiding management and conservation actions.

**FIGURE 1:**
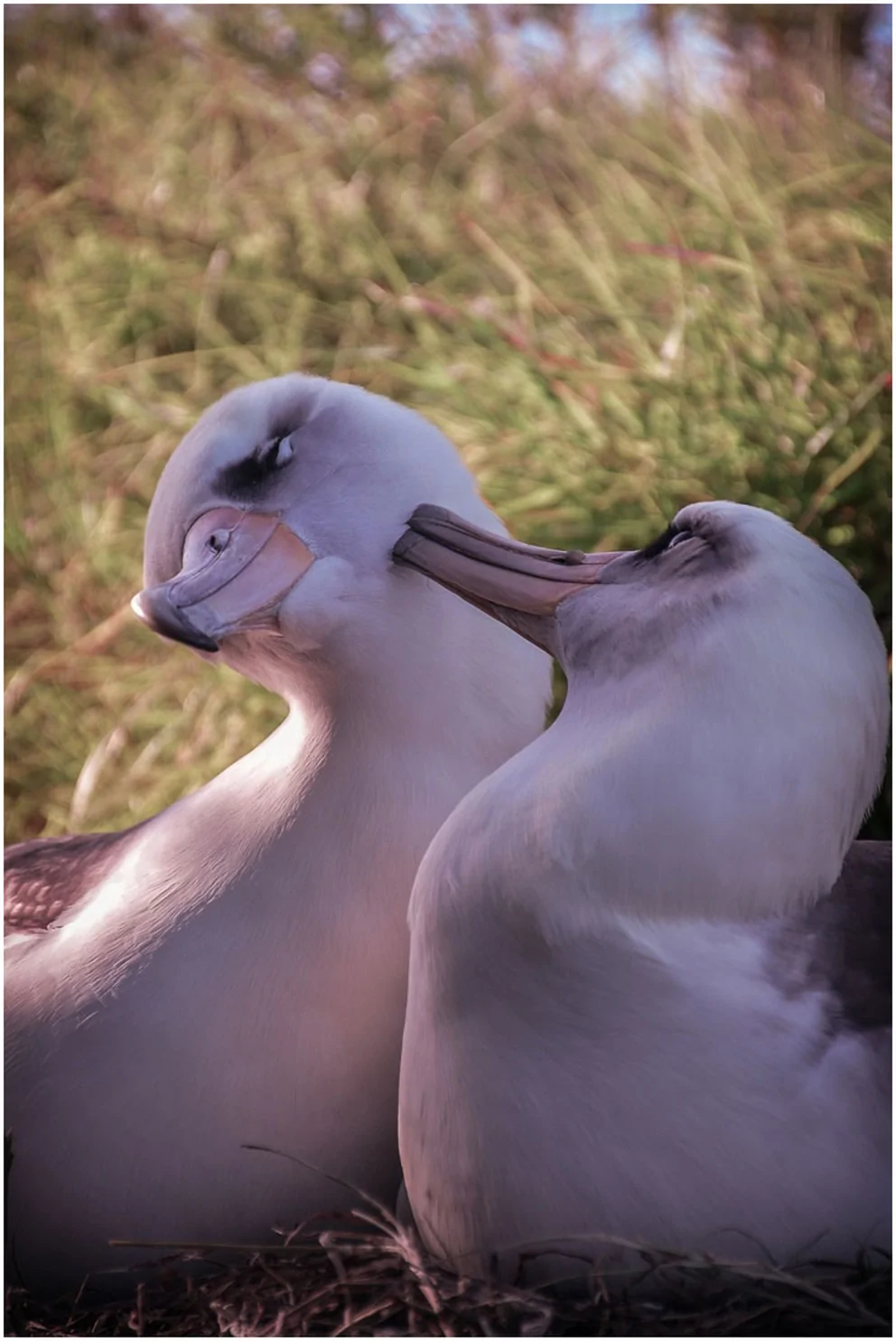
A breeding pair of Laysan Albatross ( *Phoebastria immutabilis* ) on Clarion Island.

## 2 Materials and Methods

### 2.1 Study Area

Clarion Island is one of four islands in the Revillagigedo Archipelago (18°21′ N, 114°43′ W; ~19.8 km^2^). It is the second‐largest island (6.4 × 9.7 km) in the archipelago and apparently the oldest, with an estimated age dating to the early Pliocene based on marine fossils. Situated ~1100 km offshore, Clarion Island is the most distant island from mainland Mexico (Brattstrom 2015).

Clarion Island represents the emergent portion of an oceanic shield volcano and reaches a maximum elevation of 335 m a.s.l. (Brattstrom 1990). The island has a warm, semi‐arid climate with highly seasonal rainfall, no permanent freshwater sources, and volcanic soils that support vegetation dominated by grasses, low shrubs, and cacti, with little tree cover (Comisión Nacional de Áreas Naturales Protegidas 2019). Laysan Albatrosses nest primarily in the southern and western parts of the island on bare volcanic soil and gravel in open areas with sparse vegetation. The closest nests are located ~100 m from the Mexican Navy station, the only permanent human settlement on the island.

The oceanic region around Clarion Island experiences a weak influence from the California Current and exhibits a chlorophyll‐a peak (0.10 mg·m^−3^) and low SST values (average: 26.3°C ± 1.3°C) during the first quarter of the year (Lerma et al. 2020). Overall, the waters surrounding the island are relatively stable, show minimal interannual variability, and are characterized by low productivity (average: 0.09 ± 0.02 mg·m^−3^; Lerma et al. 2020).

### 2.2 Fieldwork

We conducted fieldwork annually from January to March (when incubation and hatching occur) between 2016 and 2023. The geographic location of each nest was recorded using a handheld Garmin GPSMAP 64 GPS unit (Garmin, Olathe, USA) during an island‐wide search conducted in January, which detected all nests on the island. We marked each nest with numbered flags for monitoring and tagged adult albatrosses with alphanumeric bands (a stainless‐steel band on their right leg and a green band with one letter and a 2‐digit number on their left leg). The albatross nests were mainly located in the south and west parts of the island, in gently sloping areas near the naval base. All nests within 3 km of the naval base, accounting for about 80% of the total nests, were visited twice daily. We recorded the hatching success (eggs that hatch into live chicks) and fledging success (nestlings that survive and can fly) for each nest. To determine the causes of breeding failure at each nest, we used camera traps and direct observations; in some cases, we also relied on evidence from footprints and droppings. Chick mortality was classified as probable starvation when chicks had been observed unattended and had not been provisioned by adults for several consecutive days before death and were emaciated when found.

Two native predators, the Clarion Island whip snake ( *Masticophis anthonyi* ) and the Common Raven ( *Corvus corax* ), prey on Laysan Albatross eggs and chicks on the island (see Results). To calculate the attack rate, we used focal observations from 9 nests in 2018 and 2019, covering the period from hatching until the chicks were 2 weeks old. These continuous observation sessions took place throughout the day from 08:00 to 19:30 h, with observers rotating in three‐hour shifts. Only Clarion Island whip snakes attempted to prey on chicks during these observations.

We classified daily weather conditions as rainy, cloudy, or sunny and recorded snake approach behaviors, intrusion tactics beneath incubating parents, and the fate of affected chicks. After characterizing the initial attack patterns, an anthropogenic intervention protocol was implemented to prevent chick mortality. A snake was considered to initiate an attack when it moved directly toward the nest and attempted to slither beneath the incubating adult or approached within 10 cm of a chick. When these criteria were met, the snake was captured before it could bite or otherwise injure the chick. Snakes were captured with a herpetological hook, measured for total length, placed in a bag, weighed, and released at least 200 m from the nest. The attack rate was defined as the number of attacks observed per nest per day.

To characterize foraging behavior and identify foraging areas, GPS devices (GT‐120 loggers, i‐gotU, Miami, USA; 20–22 g, ≈1% of the body mass of the individuals) were attached to the base of three central tail rectrices of the adults during incubation (January and February) or chick‐rearing periods (February and March). Foraging trips are longer during incubation than during brooding; therefore, the GPS devices were programmed to record a position every 5 min during incubation and every minute during chick‐rearing. When an adult returned to the nest, it was recaptured to retrieve the GPS device, resulting in one foraging trip per individual. We deployed 50 GPS devices (15 in 2018, 10 in 2019, 15 in 2020, and 10 in 2022) and recovered 33 (7 in 2018, 9 in 2019, 10 in 2020, and 7 in 2022).

### 2.3 Stable Isotope Analysis

We collected blood samples for stable isotope analysis from 2018 to 2020. Samples were collected when GPS devices were retrieved after the Laysan Albatrosses returned from the foraging trip. Approximately 1 mL of blood was collected from the brachial vein, placed in a vial, and stored in a cooler to inhibit bacterial growth. In the laboratory, samples were stored frozen (−20°C) until use. We dried the samples in an oven at 60°C for 24–36 h and then ground them in an agate mortar. After grinding, the samples were sent to the Stable Isotope Facility at *Universidad Nacional Autónoma de México* for *δ* ^13^C and *δ* ^15^N analysis. Isotope ratios are reported using standard notation.

### 2.4 Data Analysis

The causes of breeding failure (given as proportions by stage [incubation or brooding]) were compared between years (2018 vs. 2019) using the Chi‐squared (χ^2^) test. To analyze attack rates (attacks·day^−1^) by Clarion Island whip snakes, we used a generalized linear mixed model. We used attack rate as the response variable, with weather conditions as a factor (three levels: sunny, cloudy, and rainy), chick age as a covariate, and nest ID as a random factor. Several distribution families (Gaussian, Poisson, and Gamma) and link functions (Gaussian, log, and inverse) were tested to determine the best fit for the data using the functions ‘lmer()’ and ‘glmer()’ from the ‘Lme4’ package in R v. 4.4.2 (R Core Team 2025).

The foraging trips were classified based on the breeding stage of the bird (incubation or brooding). For each stage, foraging variables from the tracking data were estimated using the ‘tripSplit()’ function from the ‘track2KBA’ package in R v. 4.4.2 and RStudio v. 2026.04.0+526 “Kousa Dogwood” (Beal et al. 2021; R Core Team 2025; Posit Team 2026). This function enabled us to identify individual foraging trips, separated by the return of the individual to the colony. For each trip, we calculated the maximum linear distance from the colony, total trip duration, and total distance traveled. Incomplete foraging trips (i.e., foraging trips that could not be fully tracked before the return of an individual to the colony) were excluded from all subsequent analyses. To ensure the accurate identification of individual foraging trips, a buffer area (5‐km radius) was applied around the colony to exclude periods when Laysan Albatrosses remained at the nest (Beal et al. 2021). We tested the representativeness of the tracked foraging trips separately for the incubation and brooding stages, where representativeness was defined as the extent to which the space use of tracked individuals reflected that of the population (Beal et al. 2021).

After identifying all individual foraging trips, we classified activities during each trip based on movement angles and speeds using expectation–maximization binary clustering (EMbC; Garriga et al. 2016). We identified four behavioral states: resting (low speed and low turning angles), intensive search (low speed and high turning angles), extensive search (high speed and high turning angles), and relocation (high speed and low turning angles). We used a filter to retain only low‐speed states, which are usually associated with feeding activities, including periods of rest on the water, as albatrosses can feed sitting on the water or consume carrion (Awkerman et al. 2020). We used the filtered data to calculate kernel densities and identify core use areas for feeding (KUD50). We compared average foraging parameters and core areas with those of other colonies, including Guadalupe Island and Tern Island (Henry III et al. 2021).

To determine whether foraging parameters influenced hatching success, 12 breeding attempts, including five observations from 2018 and seven from 2019, were analyzed. The response variable was binary (1 = successful hatching, 0 = failed hatching). We treated each breeding attempt as an independent observation because no individual was sampled in both years. We initially fitted binomial generalized linear models (GLMs) to evaluate the effects of breeding season (2018 or 2019), δ^13^C, δ^15^N, and foraging trip duration. However, the small sample size (*n* = 12) and distribution of hatching outcomes resulted in unstable parameter estimates and quasi‐complete or complete separation in conventional logistic models. Therefore, we used bias‐reduced logistic regression with Firth penalized maximum likelihood for the final analyses.

Before fitting models, we standardized continuous predictors to a mean of zero and unit variance. We first evaluated each predictor separately by fitting univariate models for breeding season, δ^13^C, δ^15^N, and foraging trip duration. We then evaluated models including δ^13^C in combination with each of the other predictors, as well as a model including a model with both stable isotope variables (δ^13^C and δ^15^N). The candidate model set consisted of eight models: a null model, four univariate models with breeding season, δ^13^C, δ^15^N, or foraging trip duration, and three additive models with δ^13^C and δ^15^N, breeding season, or foraging trip duration. We compared model performance using the Akaike Information criterion corrected for small sample size (AICc), calculated from the penalized log‐likelihood of the Firth models. We considered models with lower AICc values to have better support and used differences in AICc (ΔAICc) and Akaike weights to quantify relative support among candidate models. We reported parameter estimates from the selected model as regression coefficients and odds ratios (OR), with 95% confidence intervals obtained from profile penalized likelihood. Predicted probabilities of hatching success and corresponding 95% confidence intervals were calculated from the selected Firth model. All analyses were conducted in R using the logistf package.

## 3 Results

### 3.1 Breeding Success

The Laysan Albatrosses on Clarion Island exhibited no breeding success from 2016 to 2023 (Table 1). Four causes of breeding failure were identified: (1) parental abandonment/absence followed by Common Raven ( *Corvus corax* ) consumption (44%–66%), (2) nestling predation by Clarion Island whip snakes (26%–41%), (3) chick predation by ants (0%–7%), and (4) probable chick starvation (3%–7%; Table 2). During the incubation stage, all breeding failures were due to parental absence followed by raven consumption, whereas the main cause of failure during the hatching stage was predation by Clarion Island whip snakes. Although some years showed relatively high hatching success rates, all hatchlings were predated by snakes within their first few days of life. The causes of breeding failure and their effects were consistent across years (2018 vs. 2019; χ^2^ = 2.20, *p* = 0.86).

**TABLE 1:** Annual variation in colony size and breeding success of the Laysan Albatross ( *Phoebastria immutabilis* ) on Clarion Island.

Year	Colony size	Number of nests monitored	Hatching (%)	Fledglings per nest	References
1988	2	NA	NA	NA	Howell and Webb 1989
2002/03	46	15	56%	0	Wanless et al. 2009
2015/16	29	NA	NA	NA	Present study
2017/18	35	35	41%	0	Present study
2018/19	31	27	63%	0	Present study
2019/20	31	27	38%	0	Present study
2020/21	39	—	—	—	Present study
2021/22	50	41	29%	0	Present study
2022/23	39	39	16%	0	Present study

**TABLE 2:** Causes of breeding failure in the Laysan Albatross ( *Phoebastria immutabilis* ) on Clarion Island during the 2018 and 2019 breeding seasons. The table shows the number of events and the total failure percentage by stage (Inc = Incubation; Bro = Chick brooding) and year (2018 and 2019).

	2018			2019		
	Inc	Bro	Total	Inc	Bro	Total
Number of nests monitored	35	14	35	27	17	27
Parental abandonment followed by Common Raven ( *Corvus corax* ) consumption	21 100%	2 14%	23 66%	10 100%	2 29%	12 44%
Predation by Clarion whip snake (*Masticophis antonyi*)	0 —	9 64%	9 26%	0 —	11 65%	11 41%
Predation by ants	0 —	0 —	0 —	0 —	2 12%	2 7%
Probable chick starvation	0 —	1 7%	1 3%	0 —	2 12%	2 7%
Undetermined	0 —	2 14%	2 6%	0 —	0 —	0 —
Successful nests	14 40%	0 —	0 —	17 63%	0 —	0 —

### 3.2 Attack Rate by Clarion Island Whip Snakes

During focal observations, the attack rate (attacks·day^−1^) was quantified, and 190 snake attacks on nestlings were recorded across 9 nests. On their first day after hatching, the monitored chicks experienced an average of 9.8 attacks·day^−1^ (2–18 attacks·day^−1^). Generalized mixed‐model analysis showed that weather conditions and chick age significantly affected the predation rate (weather: *t* = −3.446, *p* = 0.0005; chick age: *t* = −3.231, *p* = 0.001). The predation rate was higher on sunny days than on cloudy or rainy days (Figure 1). Hatchlings faced the highest risk of predation, and the number of snake attacks decreased over time (Figure 2). Consistent with this pattern, all chicks in nests monitored without intervention were killed by snakes on the day of hatching or the following day, indicating high vulnerability to predation immediately after hatching.

**FIGURE 2:**
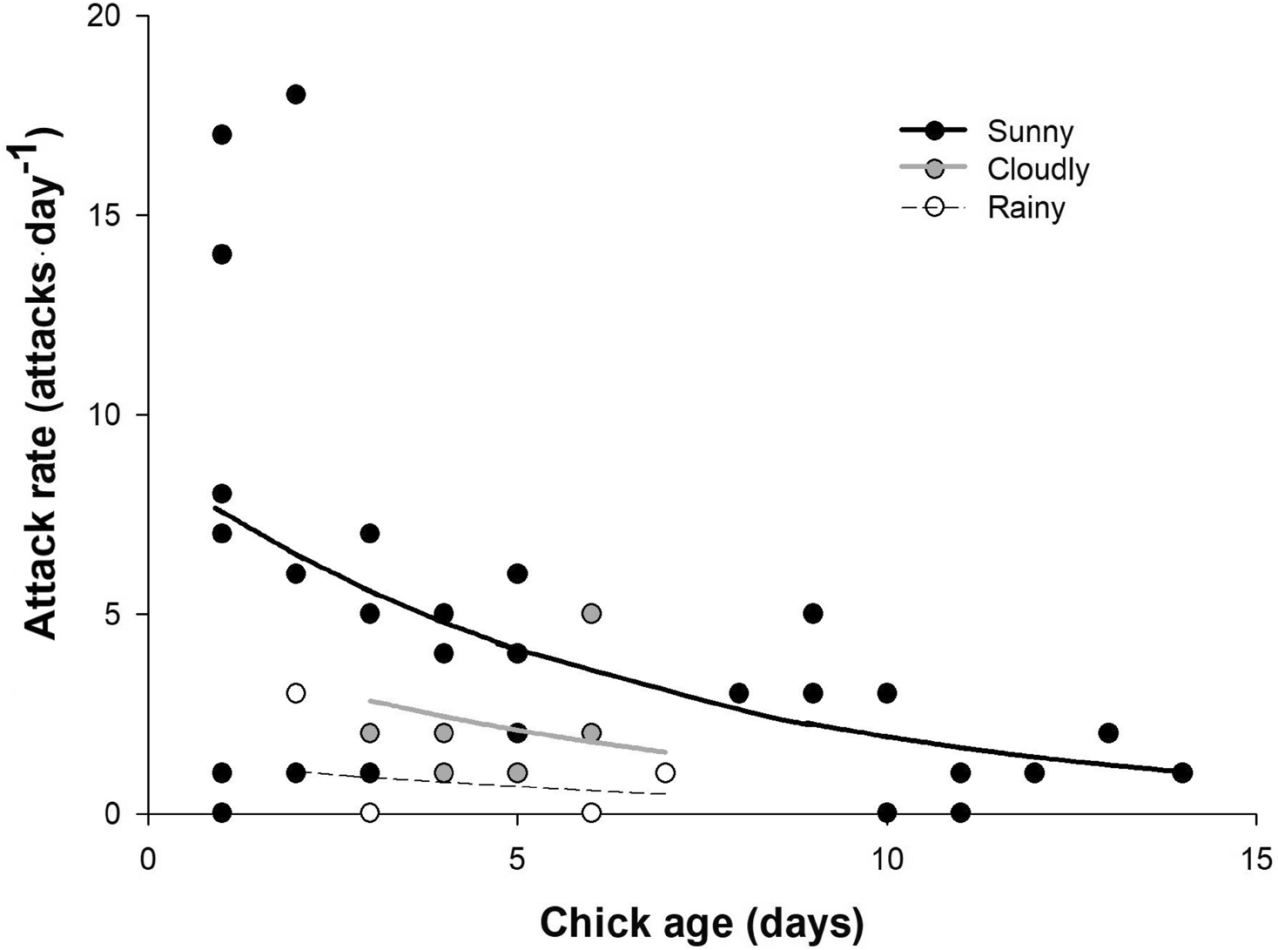
Attack rate (attacks·day^−1^) of the Clarion Island whip snake (*Masticophis antoniy*) on Laysan Albatross ( *Phoebastria immutabilis* ) chicks on Clarion Island. The figure shows the effects of weather conditions and chick age. The gamma function (log) fit is included.

### 3.3 Foraging Trips

Overall, we collected 29 complete trajectories: 11 during the incubation period and 18 during the brooding period. During the incubation period, Laysan Albatrosses traveled an average maximum distance of 3630 km from the colony, covered an average total distance of 9925 km each trip, and completed these journeys in an average of ~14.5 days. On an individual level, the Laysan Albatross that traveled the farthest spent 18 days away from the colony, moved a maximum distance of 5552 km from the colony, and covered a total distance of 14,304 km. During this period, most trips were directed northward, and the representativeness of the areas used was 90.1%.

During the brooding period, the Laysan Albatrosses traveled an average maximum distance of 291 km from the colony, covered an average total distance of 862 km, and completed their journeys within an average of 47 h. On an individual level, the Laysan Albatross that undertook the longest journey required 165 h to return to the colony, traveled a maximum distance of 1586 km, and covered a total distance of 3428 km. The representativeness of the areas used during this stage was 92%. The kernel density analysis showed that the core foraging areas of Laysan Albatrosses breeding on Clarion Island during the incubation period were primarily located within the California Current System, with a high density of use near the California coast (USA), especially between San Francisco and Los Angeles (~2000 km from the breeding colony; Figure 3). During the brooding period, Laysan Albatrosses on Clarion Island used a core area south‐southeast of the colony at a distance of ~200 km (Figure 2).

**FIGURE 3:**
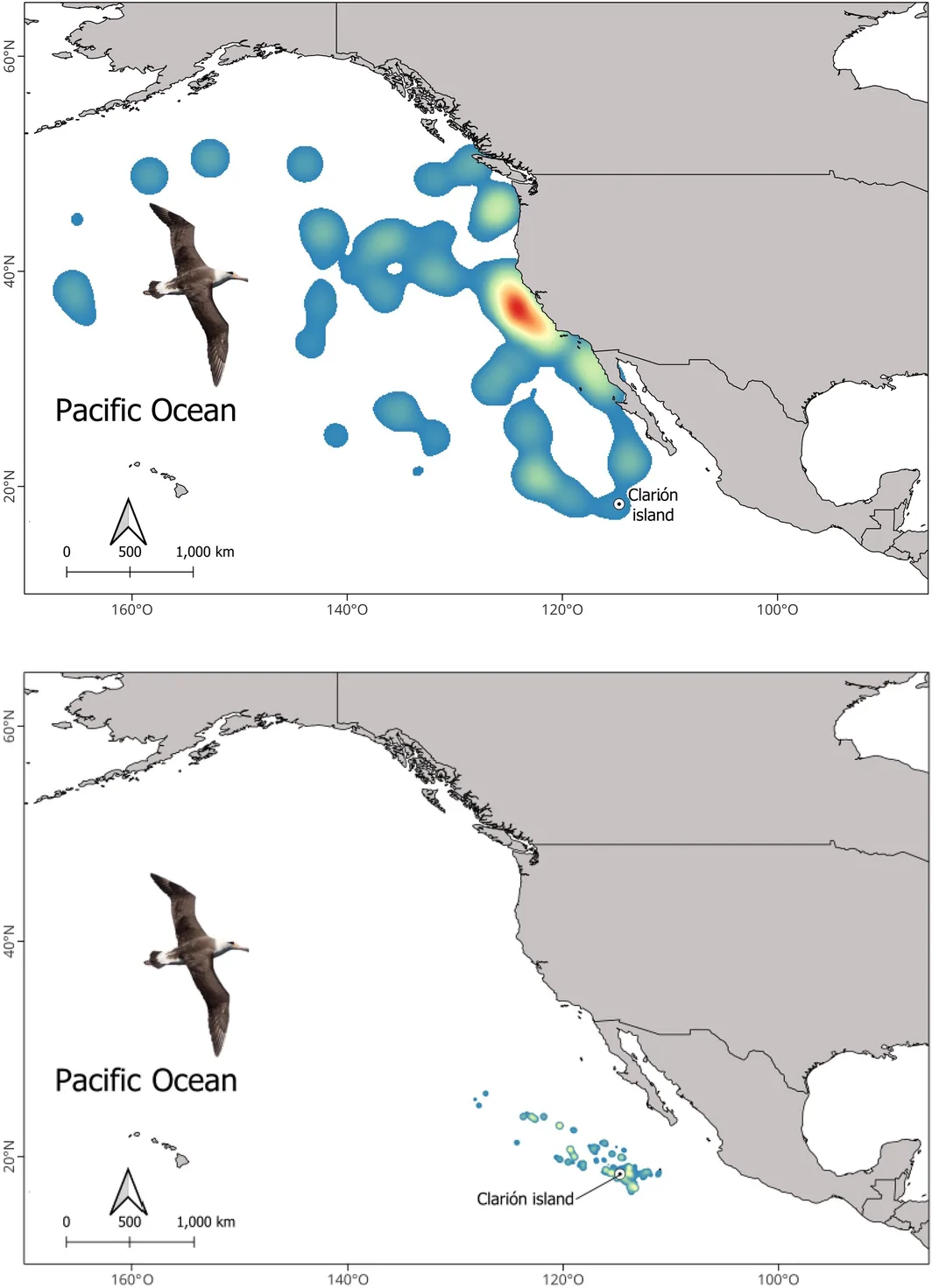
Foraging areas of Laysan Albatrosses ( *Phoebastria immutabilis* ) breeding on Clarion Island. The foraging core areas (KUD50) and kernel densities are shown for the incubation (upper panel) and brooding (lower panel) periods.

### 3.4 Influence of Foraging Parameters on Breeding Success

We evaluated hatching success for 12 breeding attempts: 10 were successful, and 2 failed. A summary of the foraging trip variables and blood stable isotope signatures for these nests is presented in Table S1. Firth bias‐reduced logistic regression revealed a negative association between δ^13^C and hatching success (β = −2.46, 95% CI: −7.61 to −0.45; likelihood‐ratio test: χ^2^ = 6.57, df = 1, *p* = 0.010), with an odds ratio of 0.09. Model comparison supported δ^13^C as the most informative predictor of hatching success among the candidate models (Table 3). The model containing δ^13^C alone had the lowest AICc (8.43), with an Akaike weight of 0.61. Models containing δ^15^N, foraging trip duration, or breeding season did not substantially improve model support (Table 3). The selected Firth model predicted a decline in the probability of successful hatching with increasing δ^13^C, although uncertainty increased toward the extremes of the observed isotopic range (Figure 4).

**TABLE 3:** Comparison of Firth logistic regression models explaining hatching success in Laysan Albatross (*Phoebastria immutabilis*) . Models are ranked by the small‐sample corrected Akaike Information Criterion (AICc). K, number of estimated parameters; logLik pen, penalized log‐likelihood; ΔAICc, difference in AICc relative to the best‐supported model; Akaike weight, relative support for each model.

Model	K	n	LogLik pen	AIC	AICc	ΔAICc	Akaike weight
δ^13^C	2	12	−1.54	7.09	8.42	0.00	0.61
δ^13^C + Trip duration	3	12	−1.37	8.74	11.74	3.32	0.11
Null	1	12	−5.12	12.24	12.64	4.21	0.07
δ^13^C + δ^15^N	3	12	−2.02	10.05	13.05	4.63	0.06
δ13C + Year	3	12	−2.41	10.83	13.83	5.40	0.04
δ^15^N	2	12	−4.35	12.71	14.04	5.61	0.04
Tri duration	2	12	−4.43	12.86	14.19	5.77	0.03
Year	2	12	−4.91	13.82	15.15	6.73	0.02

**FIGURE 4:**
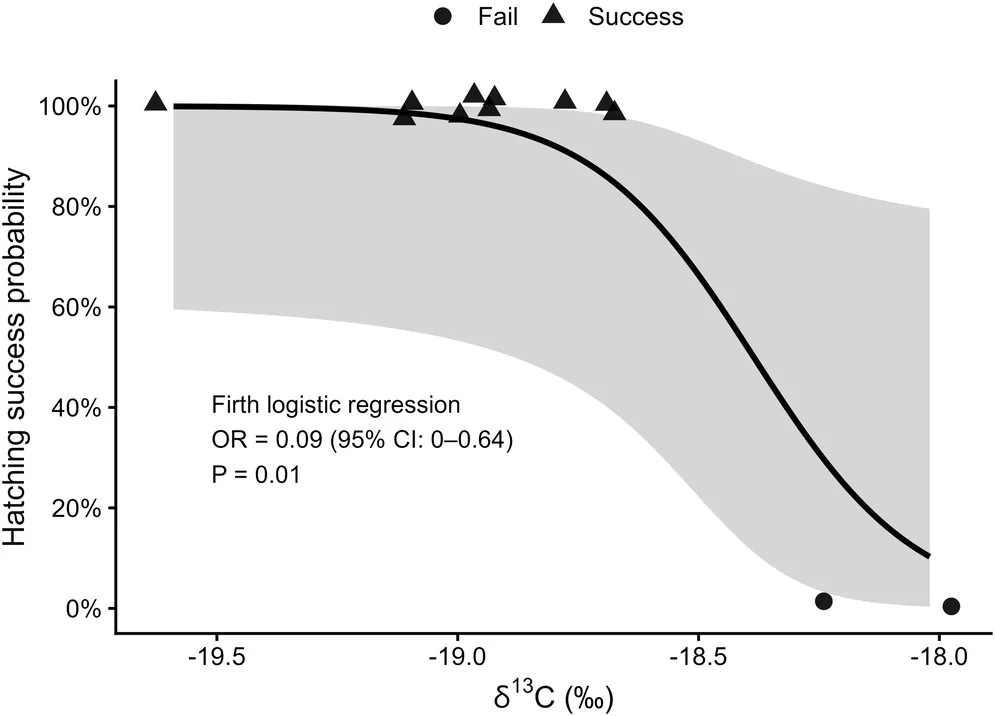
Association between red blood cell δ^13^C values and hatching success in Laysan Albatross (*Phoebastria immutabilis*). Points represent individual breeding attempts (triangles = successful hatching, circles = failed hatching), while the solid line represents the predicted probability of successful hatching from the Firth logistic regression model and the shaded area indicates the 95% confidence interval.

## 4 Discussion

The Laysan Albatross colony on Clarion Island has faced ongoing reproductive failure since at least 2003. Based on the available data, Clarion Island now hosts one of the smallest Laysan Albatross colonies in the eastern Pacific, likely due to long‐term breeding problems. The two main factors associated with this failure appear to be related to foraging ecology and intense local predation. Comparing the foraging data from Clarion Island with that from Guadalupe Island and Tern Island shows that Laysan Albatrosses on Clarion Island must travel farther to reach their feeding grounds, especially during incubation (see Table S2). During this stage, the Laysan Albatrosses of Clarion Island foraged in the California Current, mainly near the California coast (between San Francisco and Los Angeles, USA) in areas similar to those used by the Laysan Albatrosses breeding on Guadalupe Island (Henry III 2011). Additionally, the isotopic signatures of Laysan Albatrosses from Clarion Island resembled those from Guadalupe Island but differed from those of Tern Island in the central Pacific (see Figure S1). These results confirm that Laysan Albatrosses from Clarion Island mainly forage within the California Current system during incubation.

However, the geographic position of each breeding island imposes different constraints on access to these feeding grounds and may require birds to allocate their time differently between commuting and foraging. Although trip duration during incubation was relatively similar among the three colonies, the much greater distance traveled to reach the feeding grounds from Clarion Island suggests that a larger proportion of each trip may be devoted to traveling rather than to foraging. In contrast, the shorter distance from Guadalupe Island to the California Current may allow birds to spend a greater proportion of their trips in feeding areas (Henry III 2011). Thus, the geographic configuration of each colony and its distance from productive feeding grounds can strongly influence how time is allocated during foraging trips.

The high frequency of parental abandonment/absence during incubation, which increases opportunities for Common Raven egg consumption, appears to be associated with the trophic ecology of Laysan Albatrosses and the geographic location of Clarion Island. Laysan Albatrosses breeding on Clarion Island must travel 2 to 4 times farther than those in other colonies, such as those on Guadalupe Island and Tern Island, to feed in the California Current system, which limits the time they can devote to feeding. During the long incubation period (62–66 days), Laysan Albatrosses can lose considerable body mass (e.g., up to 22%; Awkerman et al. 2020). Incubation shifts last approximately 2 weeks, although either parent can incubate the egg for up to 58 days without being relieved; however, most parents abandon the egg before reaching this limit (Awkerman et al. 2020). Thus, nest attendance depends on the parental body condition. To survive, adults must maintain a body mass of at least 1600–1900 g (Awkerman et al. 2020), and Laysan Albatrosses in poorer condition are more likely to leave their eggs unattended before their partners return. Therefore, altered time allocation during foraging trips, increased foraging effort, or nutritional deficiencies may reduce parental body condition in Laysan albatrosses breeding at Clarion Island and prompt parents to prioritize survival over reproduction, leading to nest abandonment. This pattern has been documented in other seabirds, including other albatross species (Weimerskirch 1992; Croxall et al. 1999; Xavier et al. 2003).

Several studies have investigated how food affects breeding success in seabirds. Generally, these studies have concluded that food availability and quality significantly influence breeding outcomes (Croxall et al. 1999; Xavier et al. 2003; Pinaud et al. 2005; González‐Medina et al. 2018; Mills et al. 2020). The present study found that eggs from breeding Laysan Albatrosses on Clarion Island were more likely to hatch when parents had isotopic compositions less enriched in ^13^C. Thus, during incubation, differences in parental diet, as inferred from δ^13^C values, appear to be related to variation in hatching probability. Consistent with our findings, several seabird species have shown increased reproductive success when feeding on high‐quality prey (Wanless et al. 2005; Albano et al. 2011; González‐Medina et al. 2018). Although the specific prey consumed by the Laysan Albatrosses could not be identified from stable isotope signatures alone, the observed relationship between isotopic composition and hatching success provides evidence that dietary differences during incubation are associated with reproductive performance. This suggests that a limited supply of high‐quality prey may constrain reproductive performance and reduce breeding success in Laysan Albatrosses. Similar findings for Black‐browed Albatrosses ( *Thalassarche melanophris* ) and Gray‐headed Albatrosses ( *T. chrysostoma* ) have been reported by Croxall et al. (1999) and Xavier et al. (2003), respectively.

Predation by Clarion Island whip snakes was intense, with no chick surviving in the colony. The attack rate was related to chick age and the weather. The snakes were more active and made more predation attempts on warm, clear days and were much less active on cool days that were either cloudy or rainy. Weather influences predation because snakes require external heat sources to perform essential physiological processes, including digestion, which in turn controls their activities and behaviors throughout the day (Lara‐Resendiz 2020).

The concentration of snake attacks during hatching may be related to increased chemical cues at the nest. Hatching introduces new sources of organic material, including embryonic fluids, blood, and excreta, while the subsequent accumulation of feces and other organic material progressively increases the odor profile of the nest. Snakes rely strongly on chemosensory cues to locate prey, therefore, these changes may increase nest detectability. Snakes are well‐documented nest predators, and some species show higher predation rate on nestlings than on eggs (DeGregorio et al. 2014). Although the specific chemical cues that attract Clarion Island whip snakes to Laysan Albatross nests remain unknown, the abrupt increase in nest‐associated olfactory cues at hatching provides a plausible explanation for the high frequency of snake attacks observed immediately after hatching in the present study.

Widespread reproductive failure has immediate demographic effects, namely, no new individuals survive to recruit into the breeding population and replace those who die from natural or human‐induced causes. Compared with other Laysan Albatross colonies, the chronic breeding failure observed on Clarion Island is unusual and may have important implications for its colony dynamics and long‐term persistence. Although immigration commonly contributes substantially to the growth of small or recently established seabird colonies (Cairns 1992; Oro and Ruxton 2001; Young et al. 2024), long‐term colony persistence may be difficult to sustain without local recruitment. On Clarion Island, band‐recovery records revealed that two breeding individuals were banded as chicks on Whale‐Skate Island and Guadalupe Island, respectively (USGS Bird Banding Laboratory). Together with the apparent absence of local recruitment, these records suggest that the current colony size and dynamics may depend largely on immigration. Newly established seabird colonies typically rely heavily on immigrants during their initial development, with natal recruitment increasing as colonies grows (Cairns 1992; Oro and Ruxton 2001; Young et al. 2024). The persistence of the Clarion Island colony despite decades of little or no apparent local recruitment suggests an unusually prolonged dependence on immigration. In contrast, Guadalupe Island hosts the largest Laysan Albatross colony in Mexico, with 1279 reported nests in 2019, and has shown steady growth (Méndez Sánchez et al. 2022). Although only a few pairs of Laysan Albatross settled on Guadalupe and Clarion Islands in the 1980s (Agreement on the Conservation of Albatrosses and Petrels (ACAP) 2010), the growth rates and colony sizes of each island differ markedly.

Once Laysan Albatrosses select breeding sites, they return each year, even after repeated breeding failure (Young et al. 2009). For example, Laysan Albatrosses on Whale‐Skate Island returned year after year for decades despite repeated nest destruction and breeding failure caused by waves (Young et al. 2009). Indeed, breeding on the island ceased only after it was completely flooded (Young et al. 2009; Reynolds et al. 2015). The strong fidelity to breeding sites and mates exhibited by Laysan Albatrosses may enhance breeding success and facilitate reunion with partners after long periods at sea during the non‐breeding season (Reynolds et al. 2015; Awkerman et al. 2020). This limited behavioral plasticity, characterized by high site and mate fidelity and few defense mechanisms, may result in an evolutionary trap under adverse conditions (Igual et al. 2007; Reynolds et al. 2015), such as those described on Clarion Island.

## 5 Conclusions

The combination of foraging constraints and intense predation likely contributed to persistent reproductive failure for Laysan Albatrosses on Clarion Island. These factors, coupled with strong site fidelity and limited behavioral flexibility, may restrict colony growth through reproductive failure. This study provides essential baseline data on colony status and dynamics and the key threats to Laysan Albatrosses on Clarion Island and offers important insights to guide future conservation and management efforts. In particular, predator control may not be feasible because Common Ravens and Clarion Island whip snakes are native to the island, and the Clarion Island whip snake is also endemic and protected. Given that the main causes of breeding failure appear to be natural rather than anthropogenic, the need for and potential consequences of long‐term management should be carefully evaluated. Conservation actions could include the translocation of eggs or newly hatched chicks to established colonies with more favorable breeding conditions rather than indefinite intensive management to sustain breeding on Clarion Island. Additional studies evaluating the feasibility and conservation benefits of such actions are necessary before implementation.

## Author Contributions

**José Alfredo Castillo‐Guerrero:** conceptualization (lead), data curation (equal), formal analysis (equal), funding acquisition (equal), investigation (equal), project administration (equal), resources (equal), supervision (equal), validation (equal), writing – original draft (lead), writing – review and editing (equal). **Sandy Azucena Castañeda‐Castellanos:** data curation (equal), formal analysis (equal), investigation (equal), methodology (equal), writing – review and editing (equal). **Daniel Arturo Portillo‐Zavala:** data curation (equal), formal analysis (equal), investigation (equal), methodology (equal), writing – review and editing (equal). **Salvador Hernández‐Vázquez:** funding acquisition (equal), project administration (equal), resources (equal), supervision (equal), validation (equal), writing – review and editing (equal). **Guillermo Fernández:** resources (equal), supervision (equal), validation (equal), writing – review and editing (equal).

## Funding

This work was supported by the Programa de Apoyo a la Mejora en las Condiciones de Producción de los Miembros del SNII, Universidad de Guadalajara. We also acknowledge support from the Instituto de Ciencias del Mar y Limnología for the Open Access Article Publication Charge.

## Conflicts of Interest

The authors declare no conflicts of interest.

## Supporting information


**Table S1:** Foraging trip parameters and blood stable isotope signatures (mean ± SE) of Laysan Albatross ( *Phoebastria immutabilis* ) in relation to hatching success on Clarion Island.
**Table S2:** Foraging trip variables (mean ± 95% confidence interval) of the Laysan Albatross ( *Phoebastria immutabilis* ) in three colonies: Tern Island (central Pacific), Clarión Island (eastern Pacific), and Guadalupe Island (eastern Pacific). The data for Tern and Guadalupe Islands were obtained from Henry III et al. (2021).
**Figure S1:** Mean ± SD of δ^15^N and δ^13^C in the whole blood of Laysan Albatrosses ( *Phoebastria immutabilis* ) breeding in different colonies in the Pacific Ocean. The data for the Tern and Guadalupe Islands were taken from Henry III (2011); data for Midway Atoll were taken from Finkelstein et al. (2006).

**Data S1:** Supporting Information.

## Data Availability

The manuscript's data are available in the Supporting Information.
