# Supplementary material for: Breeding on a Tropical Island Proves More Difficult Than Expected: The Ongoing Breeding Failure of Laysan Albatross on Clarion Island
Source: Ecol Evol. 2026 Sep 30;16(10):e74346. doi: 10.1002/ece3.74346 (PMC13627974; doi:10.1002/ece3.74346)
Supplement: Supplementary file 1 [file ECE3-16-e74346-s002.docx]

Electronic Supplementary Material

**Breeding on a tropical island proves more difficult than expected: the ongoing breeding failure of Laysan albatross on Clarion Island**

José Alfredo Castillo-Guerrero^1^, Sandy Azucena Castañeda-Castellanos^1^, Daniel Arturo Portillo-Zavala^1^, Salvador Hernández-Vázquez^1^, and Guillermo Fernández^2*^

**Content**

**Table S1.** Foraging trip parameters and blood stable isotope signatures (mean ± SE) of Laysan Albatross (*Phoebastria immutabilis*) in relation to hatching success on Clarion Island.

**Table S2**. Foraging trip variables (mean ± 95% confidence interval) of the Laysan Albatross (*Phoebastria immutabilis*) in three colonies: Tern Island (central Pacific), Clarión Island (eastern Pacific), and Guadalupe Island (eastern Pacific). The data for Tern and Guadalupe Islands were obtained from Henry III et al. (2021).

**Figure S1.** Mean ± SD of δ^15^N and δ^13^C in the whole blood of Laysan Albatrosses (*Phoebastria immutabilis*) breeding in different colonies in the Pacific Ocean. The data for the Tern and Guadalupe Islands were taken from Henry III (2011); data for Midway Atoll were taken from Finkelstein et al. (2006).

**Table S1.** Foraging trip variables and blood stable isotope signatures (mean ± SE) of Laysan Albatross (*Phoebastria immutabilis*) in relation to hatching success on Clarion Island.

|  | Failed hatching | Successful hatching |
| --- | --- | --- |
| Trip duration (days) | 12.83 ± 2.01 | 16.20 ± 0.46 |
| Total distance (km) | 8343.33 ± 1371 | 11468.33 ± 1218 |
| Maximum distance from colony (km) | 3178.25 ± 331 | 4107.25 ± 379 |
| Time spent foraging (%) | 14.44 ± 1.50 | 15.98 ± 1.77 |
| δ^15^N | 16.76 ± 0.27 | 17.51 ± 0.07 |
| δ^13^C | -18.98 ± 0.08 | -18.13 ± 0.11 |

**Table S2**. Foraging trip variables (mean ± 95% confidence interval) of the Laysan Albatross (*Phoebastria immutabilis*) in three colonies: Tern Island (central Pacific), Clarión Island (eastern Pacific), and Guadalupe Island (eastern Pacific). The data for Tern and Guadalupe Islands were obtained from Henry III et al. (2021).

| **Trip characteristics by stage** | **Clarión Island** | **Guadalupe Island** | **Tern Island** |
| --- | --- | --- | --- |
| **Incubation** |  |  |  |
| Distance to colony (km) | 3,630  (3,009–4,251) | 861  (601–1,196) | 1,646  (1,538–1,774) |
| Total distance traveled (km) | 9,925  (6,953–12897) | 4,633  (3,413–6,530) | 6,444  (5,455–7,406) |
| Duration (days) | 14.5  (12.0–17.0). | 12.1  (9.5–15.0) | 16.2  (14.0–18.9) |
| **Brooding** |  |  |  |
| Distance to colony (km) | 291  (134–447) | 93  (58–129) | 164  (125–210) |
| Total distance traveled (km) | 862  (523–1,201) | 554  (433–722) | 821  (548–1,049) |
| Duration (days) | 1.9  (1.3–2.6). | 2.4  (2.0–2.8) | 2.7  (2.2–2.9) |

**Figure S1**. Mean ± SD of δ^15^N and δ^13^C in the whole blood of Laysan Albatrosses (*Phoebastria immutabilis*) breeding in different colonies in the Pacific Ocean. The data for the Tern and Guadalupe Islands were taken from Henry III (2011); data for Midway Atoll were taken from Finkelstein et al. (2006).
