# Supplementary material for: Breeding on a Tropical Island Proves More Difficult Than Expected: The Ongoing Breeding Failure of Laysan Albatross on Clarion Island
Source: Ecol Evol. 2026 Sep 30;16(10):e74346. doi: 10.1002/ece3.74346 (PMC13627974; doi:10.1002/ece3.74346)
Supplement: Supplementary file 2 [file ECE3-16-e74346-s001.zip › Resumen_R2.docx]

**Resumen**

En la década de 1980, el albatros de Laysan (*Phoebastria immutabilis*) amplió su área de distribución reproductiva hacia el Pacífico oriental y estableció colonias en varias islas de México. El crecimiento de las colonias ha variado entre estas islas, lo que probablemente refleja diferencias en las tasas de inmigración y en las condiciones locales, como la disponibilidad de alimento y el riesgo de depredación, factores que pueden influir en el éxito reproductivo y en la dinámica poblacional. Evaluamos el desempeño reproductivo de los albatros de Laysan en la isla Clarión (2016–2023) y su relación con la ecología de forrajeo y la depredación local. Monitoreamos el éxito reproductivo mediante revisiones de nidos y cuantificamos la tasa de depredación mediante observaciones focales. Se obtuvieron las trayectorias de los viajes de alimentación mediante dispositivos GPS y se analizaron muestras de sangre para determinar la composición isotópica de nitrógeno (δ¹⁵N) y carbono (δ¹³C). Durante el período de estudio, la colonia experimentó un fracaso reproductivo total. Nuestros resultados sugieren que dos factores probablemente contribuyeron a este desenlace: (1) limitaciones en el forrajeo y (2) altas tasas de depredación. La ubicación remota de la isla Clarión obligó a los albatros de Laysan a recorrer largas distancias hasta las zonas de alimentación, lo que potencialmente redujo el tiempo disponible para capturar presas de alta calidad. Esto pudo haber influido en sus firmas de δ¹³C, que mostraron una asociación con el éxito de eclosión. Además, las crías sufrieron niveles de depredación extremadamente altos. En conjunto, las aparentes limitaciones alimentarias y la intensa depredación local obstaculizaron tanto el éxito reproductivo como el reclutamiento local. Estos factores podrían explicar el crecimiento limitado de la colonia de Clarión en las últimas décadas. Este estudio aporta datos de referencia sobre el estado de la colonia y sus principales amenazas, información fundamental para la planificación de estrategias de conservación y manejo.
